# Insomnia

**Published:** 1899-07-22

**Authors:** 


Vol. XXVI.—No. 669.] Edited by Sir Henry Burdett, K.C.B., and Solomon C. Smith, M.D., M.R.C.P. [July 22, 1899.

### Insomnia.

The very interesting study of hypnotics to which Professor Bradbury has devoted the Croonian lectures lately delivered before the Royal College of Physicians serves to show how complicated is the problem to be solved by the physician who would satisfactorily treat a case of sleeplessness. There is, indeed, an initial and an abiding difficulty which overhangs the whole subject, namely, that we do not know, and that Professor Bradbury is unable to tell us, what is the cause of normal sleep. In the first lecture of the series he gives very succinctly a sketch of the several theories which have been held on the subject, but he is unable to state with anything like definiteness what is the condition of the brain cells, or of the fluid by which they are fed, which leads to the production of sleep. It seems fair to believe that, whatever difference there may be in the histology of the nerve cell and in its blood supply during the waking and the sleeping states, the determination of these states is ultimately due to some change in the chemical environment of the neuron, or the chemical metabolism within it, but we are as far as ever from being able to define what is that chemical change which wraps the cell in drowsiness.

In some way—direct or roundabout, according to the drug used—it is possible to influence the brain cells in such a manner that sleep is produced, but we have no reason to believe that by the operation of any one of these drugs the same chemical change, the same kind of cell metabolism, is set up as is the starting-point of natural sleep, similar as may be the phenomena which result. Moreover, the drugs by which sleep can be produced do not unfortunately act upon the cerebral cells alone; their administration often produces effects which are not found accompanying or following natural sleep, and at the best their administration leads to a condition which is closely allied to, if not identical with, a pathological state. Thus, in the search for a hypnotic we have not to be guided entirely by which is the most reliable, but also, and more especially, by which is the least toxic. The treatment of sleeplessness, however, is not merely a matter of discovering a suitable hypnotic. The very first essential is that, if possible, the causes of insomnia shall be discovered and removed, and they are legion. First among them are what may be termed irritative causes; the irritation of an eczema, the restraint of bandages or of position after a surgical operation, coldness of the feet, vesical affections, cough, and a score of other troubles are all conditions which, like pain, lead to sleeplessness, and, like pain, should, if possible, be removed before recourse is had to direct hypnotics. Then there are toxic causes. Alcoholism and nicotism; the toxæmia accompanying various febrile diseases and various disorders of the digestive tract; the condition produced by various beverages, such as tea, coffee, &c., and by opium-eating or the taking of cocaine, may all destroy the natural tendency to sleep. Many psychical causes may also have the same effect, even such simple ones as mere change in the ordinary routine of life, as in travelling, and as is often seen in nurses when changing from day to night duty, and *vice versâ*. Now, although in regard to all these causes the first thing to do is to remove them, that is often not enough. The brain gets into bad habits, and sleep does not at once return even when the cause of its flight has been removed. It is in these cases that hypnotics are often not only useful, but necessary, for by their aid alone can the habit of sleeplessness be broken.

Although it cannot be said that there is any one drug which is absolutely safe, both experimental investigation and clinical observation show that paraldehyde stands in the first rank. Then there are chloramide and chloralose, which, and especially the former, are safer, although slower in action, than chloral hydrate. Sulphonal, again, is a valuable drug for the purpose; but, on the whole, the bromides may be regarded as the least harmful, and may best be resorted to in the first instance when a hypnotic is required.

Sleeplessness from overwork, and especially from literary work, requires rest and change of air and scene, while in the insomnia of Bright's disease eliminants, such as aperients, and, if they fail, chloral hydrate, may be given, the reduction of blood pressure which this drug produces being beneficial rather than otherwise in this complaint. Erythrol tetranitrate again often acts like a charm in this disease by reducing arterial tension. In neuralgia phenazonum or phenacetin may be of great value, but as a rule where pain is the causal factor of insomnia opium is the best general remedy.

The general measures which conduce to sleep are numerous, and the hypnotic drugs at our disposal increase in number every day, each with its own special powers and its own particular dangers. Of the measures which conduce to sleep, matters of diet and nursing, and what may be called "management," we cannot be too studious, for by their aid much may be done to avoid the use of drugs. But if drugs must be employed, now more than ever is it necessary that the physician should make a careful choice, for with better weapons than of old he is expected to take better aim.

